# Sex-related differences in smear-positive pulmonary tuberculosis patients in Kuala Lumpur, Malaysia: Prevalence and associated factors

**DOI:** 10.1371/journal.pone.0245304

**Published:** 2021-01-08

**Authors:** Norfazilah Ahmad, Mazni Baharom, Azimatun Noor Aizuddin, Rohaya Ramli

**Affiliations:** 1 Department of Community Health, Faculty of Medicine, Universiti Kebangsaan Malaysia, Bandar Tun Razak, Kuala Lumpur, Malaysia; 2 Tuberculosis and Leprosy Unit, Health Department of Federal Territory Kuala Lumpur & Putrajaya, Ministry of Health Malaysia, Kuala Lumpur, Malaysia; Jamia Hamdard, INDIA

## Abstract

Smear-positive pulmonary tuberculosis (PTB) is more infectious compared to smear-negative PTB and have great significance for epidemiology and infection control. The prevalence of smear-positive PTB rarely affects males and females equally. Hence, we aimed to identify the sex-related differences in the prevalence of smear-positive PTB and its associated factors in Kuala Lumpur, Malaysia. A cross-sectional study was conducted using data from the National Tuberculosis Information System (TBIS) from 1 January, 2015, to 31 December, 2019. The study population was selected using simple random sampling from the list of registered PTB patients in TBIS. The criteria for inclusion were all Malaysian adults aged ≥18 years residing in Kuala Lumpur and registered as PTB in TBIS. Factors associated with smear-positive PTB in male and female patients were determined using multiple logistic regression analysis. Overall prevalence of smear-positive PTB was 68.6%, and male patients predominated (71%). The male:female prevalence ratio of smear-positive PTB was 2.4:1. Male patients who worked as machine operators and elementary workers (adjusted odds ratio (aOR) 2.23, 95% confidence interval (CI) 1.24–4.02, *p* = 0.007), were self-employed (aOR 2.58, 95% CI 1.46–4.56, *p* = 0.001), lived in a residence categorized as ‘other’ (aOR 2.49, 95% CI 1.28–4.86, *p* = 0.007) and were smokers (aOR 1.37, 95% CI 1.01–1.87, *p* = 0.045) had higher odds for smear-positive PTB. Meanwhile, female patients with diabetes mellitus had higher odds for smear-positive PTB (aOR 1.92, 95% CI 1.05–3.54, *p* = 0.035), while female patients who were healthcare workers had lower odds (aOR 0.33, 95% CI 0.12, 0.94, *p* = 0.039). The prevalence of smear-positive PTB is higher in males compared to females. The factors associated with smear-positive PTB differed based on sex. The current TB control program, especially on smear-positive PTB, should likely be strategized and stratified by sex.

## Introduction

Tuberculosis (TB) is an infectious disease caused by the tubercle bacilli called *Mycobacterium tuberculosis* (*MTb*). According to the World Health Organization (WHO), approximately a quarter (1.7 billion) of the world population are latently infected with *MTb*, where they have a 5–10% lifetime risk of becoming sick or infectious with TB [1]. In 2019, TB caused around 10 million new cases worldwide, with an estimated global incidence of 130 cases per 100 000 population. Of all TB cases, 84% are pulmonary TB (PTB), among which 57% are bacteriologically confirmed [1]. Malaysia is a moderate burden TB country with an incidence of 92 per 100 000 population [1]. The most common type of TB in Malaysia was PTB (82.2%), and 81.2% of new PTB cases in 2018 were smear-positive PTB [2]. The evidence shows that smear-positive PTB is more infectious compared to smear-negative PTB. The transmission risk is higher if the index case is sputum smear–positive, and is corresponds to the respiratory secretion bacillary density [3].

The burden of TB varies enormously among countries, and it also rarely affects males and females equally. Most studies conducted globally have revealed that the highest burden of TB is among males [4, 5]. A similar male predominance of an approximately 2:1 ratio was also observed for PTB [6] and smear-positive PTB [7] in Southern Mexico and Iran, respectively. Nevertheless, there were also studies recorded higher smear-positive PTB prevalence among females, such as in Quetta city [8] and Hyderabad city [9] in Pakistan. These sex-related variations may reflect the difference in biological [10], epidemiological and socioeconomic characteristics, and cultural barriers in accessing healthcare services [11]. Findings from research exploring on sex-related differences in risk factor of PTB [5–7] invariably differ according to the local setting of the PTB patient’s population. Therefore, it is appropriate to perform sex-difference assessment and analysis in the local context as an evidence-based for strengthening the National Strategic Plan of TB control program as recommended by WHO [11] and United Nations Development Programme [12]. These data are vital for planning and executing strategic interventions to address the sex dimensions of TB [12].

There is a limited local study looking at factor associated with smear-positive PTB in the general population. In Malaysia, most of the studies conducted in a hospital setting and focus on particular groups such as HIV positive patients [13], diabetes mellitus [14], healthcare worker [15] and inmates [16]. On top of these, to our knowledge, there is no local study looking at the sex-related differences in smear-positive PTB and its associated factors. Hence, the present study aimed to identify the sex-related differences in the prevalence of smear-positive PTB and its associated factors in Kuala Lumpur, Malaysia.

## Materials and methods

### Ethical approval

This study was approved by the Medical Research and Ethics Committee of the Ministry of Health Malaysia (NMRR-20-91-52679) and from the Universiti Kebangsaan Malaysia Research Ethics Committee (UKM REC) (UKM PPI/111/8/JEP-2020-168).

### Study setting

Kuala Lumpur is the capital city of Malaysia, and has the highest population density in the country. In 2019, the estimated population of Kuala Lumpur was 1.78 million, with 916 100 males (51.4%) and 864 700 females (48.6%) [17]. In 2019, the population density was 7737 persons per km^2^ [17]. Briefly, the study area includes four districts that serve as administrative subdivisions under the authority of the Kuala Lumpur and Putrajaya Federal Territory Health Department.

### Study design and population

This was a cross-sectional study involving patients with smear-positive PTB in Kuala Lumpur. The study population was patients diagnosed and registered as PTB in the National Tuberculosis Information System (TBIS) from 1 January, 2015, to 31 December, 2019. The TBIS contains information on all TB cases notified to the District Health Office. It is mandatory to notify TB cases once any medical officers make a diagnosis in both private and public healthcare facilities. Details on the TBIS have been published elsewhere [18].

The inclusion criteria were all Malaysian adults aged ≥18 years residing in Kuala Lumpur and registered as new PTB cases in the TBIS. Patients diagnosed as PTB with extrapulmonary organ involvement, patient who is registered as re-treated cases (referring to the previously treated patient which the patient received 1 month or more anti-TB drugs in the past, mainly include relapses, treatment after failure, or loss to follow-up on a first-line treatment regimen) [19] and patients with incomplete data in the TBIS were excluded. The sample size was calculated using Power and Sample Size software [20] and in reference to Jimenez-Corona et al. [6] with power of 90% and a confidence interval (CI) of 95%. A minimum sample size of 1157 was obtained. The study population was selected using simple random sampling from the list of registered PTB cases in the TBIS in a 5-year duration (2015–2019).

### Study instruments

The data retrieved from the TBIS spanned 1 January, 2015, to 31 December, 2019. The retrieved information included sociodemographic data (age, race, residential location), socioeconomic data (education level, type of residence, occupation, income level), clinical characteristics (smoking, DM and HIV status) and environmental factors (overcrowded household, healthcare worker (HCW) status).

### Outcome variables

The outcome was smear-positive PTB, and was defined as a PTB patient with at least one or more initial sputum smear examinations (direct smear microscopy) positive for acid-fast bacilli (AFB), or with one sputum specimen positive for AFB and radiographic abnormalities consistent with active PTB, or with one sputum specimen positive for AFB and culture positive for *MTb* [19]. Smear-negative PTB was defined as a PTB patient with at least three negative results in direct smear sputum microscopy but with radiographic results suggestive of active TB or sputum culture positive for *MTb* [19].

### Independent variables

Age was categorized into 18–38-, 39–59-, 60–80-, and ≥81-year age groups. Sex was classified as male or female. Race was considered inherited from parents’ assignments based on the Malaysian identity card (Malay, Chinese, Indian, other). Residential location was categorized based on the territories of the Kuala Lumpur and Putrajaya Federal Territory Health Department, namely Cheras, Kepong, Lembah Pantai and Titiwangsa.

Education level was categorized based on the highest education level achieved, and was categorised as primary (Standard 1–6), secondary (Form 1–5), tertiary (certificates, diplomas or academic degrees) or no formal education. Type of residence was categorised into low-cost, medium- to high-cost, and other. Low-cost residences were defined as low-cost flats and residence under the government housing programs (*Projek Perumahan Rakyat*, *PPR*) [18]. Medium- to high-cost residences were defined as apartments, condominiums, terrace houses and bungalows. The ‘other’ group included residence in homeless shelters, shop lots in commercial areas, senior citizen care centres, or government institutions, i.e. prisons. Occupation was categorised based on the classifications by the Ministry of Human Resources Malaysia [21]: professionals (tertiary education leading to a university or postgraduate university degree); technicians, associate professionals and skilled workers (tertiary education leading to a Malaysian Skills Certificate); clerical, service and sales support workers (secondary or post-secondary education); machine operators and elementary workers; self-employed; and unemployed [21]. Pensioners, housewives and students were classified as ‘other’. Household income was considered low, middle and high when the median household income was Ringgit Malaysia (RM) 3000 and below (≤RM 3000), RM 3001–13 148, and ≥RM 13 149, respectively [17].

Smoking status was self-reported. Smokers were categorised as ‘yes’ and non-smokers were categorised as ‘no’. For DM status, patients with underlying DM were classified as ‘yes’, and those without DM were classified as ‘no’. HIV status was based on the result of HIV rapid test screening after the TB diagnosis.

Overcrowded household was classified as ‘yes’ or ‘no’ [22]. Overcrowded is defined as >5 inhabitants living in a low-cost flat (60–70 m^2^) and >6 inhabitants living in a medium- or high-cost residential property (high-rise > 93 m^2^ or terrace house > 93 m^2^ and other types of residences). For HCW status, a patient whose occupation related to healthcare was categorized as ‘yes’ and that unrelated to healthcare was categorized as ‘no’.

### Statistical analysis

Data analyses were conducted using Statistical Package for the Social Sciences (SPSS) version 21. Descriptive analysis was performed to determine the mean and standard deviation (SD) for continuous data, while categorical data were presented as frequency (*n*) and percentage (%). The association between the independent variables and smear-positive PTB was determined using simple logistic regression to estimate the crude odds ratio (cOR) and its 95% confidence interval (CI). Multiple logistic regression with variables’ *p* < 0.05 was performed to obtain the adjusted OR (aOR) and 95% CI.

## Results

### Study population characteristics

Of 1211 PTB cases between 1 January, 2015, and 31 December, 2019, in Kuala Lumpur, 69% (837) were male, and 374 (31%) were female, yielding a male to female (M:F) ratio of 2.3:1. Table 1 shows the study population sociodemographic, socioeconomic, clinical and environmental characteristics stratified by sex. Female patients had a lower median age [39.0 (SD 18.6) years] compared to male patients [48.0 (SD 16.0) years]. The distribution was almost equal in the four residential areas, and 51.7% of patients were Malay. The majority of patients had at least secondary school education (60.0%), were low-income (94.1%) and were not HCW (98.2%).

**Table 1:** The baseline characteristics of PTB cases.

	PTB cases		
Characteristic	Male (*n* = 837)	Female (*n* = 374)	Total (*n* = 1211)
	*n* (%)	*n* (%)	*n* (%)
***Sociodemographic characteristics***			
**Age (years) (median (SD))**	48 (16)	39 (18.6)	46 (17)
**Age group (years)**			
18–38	270 (32.3)	184 (49.2)	454 (37.5)
39–59	370 (44.2)	108 (28.9)	478 (39.5)
60–80	185 (22.1)	72 (19.3)	257 (21.2)
≥81	12 (1.4)	10 (2.7)	22 (1.8)
**Race**			
Malay	409 (48.9)	217 (58)	626 (51.7)
Chinese	295 (35.2)	98 (26.2)	393 (32.5)
Indian	102 (12.2)	45 (12)	147 (12.1)
Other	31 (3.7)	14 (3.7)	45 (3.7)
**Residential location**			
Cheras	200 (23.9)	68 (18.2)	268 (22.1)
Kepong	229 (27.4)	106 (28.3)	335 (27.7)
Lembah Pantai	214 (25.6)	82 (21.9)	296 (24.4)
Titiwangsa	194 (23.2)	118 (31.6)	312 (25.8)
***Socioeconomic characteristics***			
**Education level**			
No formal education	75 (9)	39 (10.4)	114 (9.4)
Primary school	77 (9.2)	19 (5.1)	96 (7.9)
Secondary school	531 (63.4)	196 (52.4)	727 (60)
Tertiary education	154 (18.4)	120 (32.1)	274 (22.6)
**Type of residence**			
Low-cost	376 (44.9)	171 (45.7)	547 (45.2)
Medium–high-cost	380 (45.4)	184 (49.2)	564 (46.6)
Other	81 (9.7)	19 (5.1)	100 (8.3)
**Occupation**			
Professional and manager	34 (4.1)	25 (6.7)	59 (4.9)
Technicians, associate professionals, skilled workers and armed forces	46 (5.5)	30 (8)	76 (6.3)
Clerical, service, sales and support workers	169 (20.2)	76 (20.3)	245 (20.2)
Machine operators and elementary workers	137 (16.4)	7 (1.9)	144 (11.9)
Self-employed	150 (17.9)	48 (12.8)	198 (16.4)
Unemployed	208 (24.9)	75 (20.1)	283 (23.4)
Other	93 (11.1)	113 (30.2)	206 (17)
**Income level (RM)**			
Low (≤3000)	792 (94.6)	348 (93.0)	1140 (94.1)
Middle (3001–13 148)	44 (5.3)	25 (6.7)	69 (5.7)
High (≥13 149)	1 (0.1)	1 (0.3)	2 (0.2)
***Clinical characteristics***			
**Smoking**			
Yes	452 (54)	18 (4.8)	470 (38.8)
No	385 (46)	356 (95.2)	741 (61.2)
**DM**			
Yes	188 (22.5)	68 (18.2)	256 (21.1)
No	649 (77.5)	306 (81.8)	955 (78.9)
**HIV status**			
Positive	75 (9)	8 (2.1)	83 (6.9)
Negative	762 (91)	366 (97.9)	1128 (93.1)
***Environmental characteristics***			
**Overcrowded household**			
Yes	107 (12.8)	39 (10.4)	146 (12.1)
No	730 (87.2)	335 (89.6)	1065 (87.9)
**HCW**			
Yes	6 (0.7)	16 (4.3)	22 (1.8)
No	831 (99.3)	358 (95.7)	1189 (98.2)

### Prevalence and characteristics of smear-positive PTB patients stratified by sex

The prevalence of smear-positive PTB was 68.6%. Most of the cases were male (71%) with a M:F prevalence ratio of smear-positive PTB of 2.4:1. Table 2 shows that male patients had eight factors with higher prevalence and cOR for smear-positive PTB as compared to each respective reference group. The prevalence of smear-positive male patients residing in Lembah Pantai was 76.2% (cOR 1.65, 95% CI 1.07, 2.53, *p* = 0.023); 73.6% had at least secondary school education (cOR 1.64, 95% CI 1.12, 2.40, *p* = 0.011) and 85.2% lived in ‘other’ residences (cOR 2.44, 95% CI 1.27, 4.68, *p* = 0.007). A total of 78.7% of male patient were self-employed (cOR 2.78, 95% CI 1.58, 4.91, p < 0.001), 77.4% were machine operators or elementary workers (cOR 2.58, 95% CI 1.46, 4.58, p = 0.001), 70.2% were unemployed (cOR 1.78, 95% CI 1.07, 2.95, p = 0.026) and 69.2% worked as clerical, services, sales and support workers (cOR 1.70, 95% CI 1.01, 2.87, p = 0.048). All of them were diagnosed as smear-positive PTB. Up to 74.1% of smear-positive male patients were smokers (cOR 1.46 (95% CI 1.08, 1.97, *p* = 0.013).

**Table 2:** Characteristics of smear-positive PTB and simple logistic regression analysis among male PTB patients.

			Male patients		
Characteristic	Smear-positive PTB (*n* = 590) 81.6%	Smear-negative PTB (*n* = 247) 50.6%	Crude OR^c^ (95% CI)	χ^2^ (df)^a^	*p-*value
***Sociodemographic characteristics***	***n* (%)**	***n* (%)**			
**Age group (years)**					
18–38	184 (68.1)	86 (31.9)	1		
39–59	276 (74.6)	94 (25.4)	1.37 (0.97, 1.91)	3.198 (1)^b^	0.074
60–80	123 (66.5)	62 (33.5)	0.93 (0.62, 1.38)	0.138 (1)^b^	0.710
≥81	7 (58.3)	5 (41.7)	0.65 (0.20, 2.12)	0.500 (1)^b^	0.480
**Race**					
Malay	287 (70.2)	122 (29.8)	1		
Chinese	204 (69.2)	91 (30.8)	0.95 (0.69, 1.32)	0.084 (1)^b^	0.772
Indian	79 (77.5)	23 (22.5)	1.46 (0.88, 2.43)	2.112 (1)^b^	0.146
Other	20 (64.5)	11 (35.5)	0.77 (0.36, 1.66)	0.435 (1)^b^	0.510
**Residential location**					
Cheras	132 (66)	68 (34)	1		
Kepong	161 (70.3)	68 (29.7)	1.22 (0.81, 1.83)	0.913 (1)^b^	0.339
Lembah Pantai	163 (76.2)	51 (23.8)	1.65 (1.07, 2.53)	5.177 (1)^b^	**0.023**
Titiwangsa	134 (69.1)	60 (30.9)	1.15 (0.75, 1.76)	0.424 (1)^b^	0.515
***Socioeconomic characteristics***					
**Education level**					
No formal education	52 (69.3)	23 (30.7)	1.33 (0.74, 2.40)	0.891 (1)^b^	0.345
Primary school	50 (64.9)	27 (35.1)	1.09 (0.62, 1.92)	0.084 (1)^b^	0.772
Secondary school	391 (73.6)	140 (26.4)	1.64 (1.12, 2.40)	6.535 (1)^b^	**0.011**
Tertiary education	97 (63)	57 (37)	1		
**Type of residence**					
Low-cost	264 (70.2)	112 (29.8)	1		
Medium–high-cost	257 (67.6)	123 (32.4)	0.89 (0.65, 1.21)	0.588 (1)^b^	0.443
Other	69 (85.2)	12 (14.8)	2.44 (1.27, 4.68)	7.194 (1)^b^	**0.007**
**Occupation**					
Professionals and managers	22 (64.7)	12 (35.3)	1.38 (0.61, 3.12)	0.611 (1)^b^	0.435
Technicians, associate professionals and skilled workers	28 (60.9)	18 (39.1)	1.17 (0.57, 2,41)	0.190 (1)^b^	0.663
Clerical, service, sales and support workers	117 (69.2)	52 (30.8)	1.70 (1.01, 2.87)	3.914 (1)^b^	**0.048**
Machine operators and elementary workers	106 (77.4)	31 (22.6)	2.58 (1.46, 4.58)	10.505 (1)^b^	**0.001**
Self-employed	118 (78.7)	32 (21.3)	2.78 (1.58, 4.91)	12.533 (1)^b^	**<0.001**
Unemployed	146 (70.2)	62 (29.8)	1.78 (1.07, 2.95)	4.947 (1)^b^	**0.026**
Other	53 (57)	40 (43)	1		
**Income level (RM)**					
Low (≤3000)	561 (70.8)	231 (29.2)	1		
Middle (3001–13 148)	29 (65.9)	15 (34.1)	0.80 (0.42, 1.51)	0.485 (1)^b^	0.486
High (≥13 149)	0 (0)	1 (100)	0 (0)	0 (1)^b^	1.000
***Clinical characteristics***					
**Smoking**					
Yes	335 (74.1)	117 (25.9)	1.46 (1.08, 1.97)	6.181 (1)	**0.013**
No	255 (66.2)	130 (33.8)	1		
**DM**					
Yes	142 (75.5)	46 (24.5)	1.39 (0.96, 2.01)	2.948 (1)	0.086
No	448 (69)	201 (31)	1		
**HIV**					
Yes	48 (64)	27 (36)	0.72 (0.44, 1.19)	1.657 (1)	0.198
No	542 (71.1)	220 (28.9)	1		
***Environmental characteristics***					
**Overcrowded household**					
Yes	78 (72.9)	29 (27.1)	1.15 (0.73, 1.81)	0.341 (1)	0.559
No	512 (70.1)	218 (29.9)	1		
**HCW**					
Yes	4 (66.7)	2 (33.3)	0.84 (0.15, 4.60)	0.042 (1)	0.837
No	586 (70.5)	245 (29.5)	1		

^a^Likelihood ratio (LR) test

^b^Wald test.

^c^Crude OR using simple logistic regression; bolded, *p* < 0.05; 1 = reference

*Type of residence categorised as ‘other’ include residence in homeless shelters, shop lots in commercial areas, senior citizen care centres, or government institutions, i.e. prisons.

Table 3 shows that female patients had two factors with higher prevalence and cOR for smear-positive PTB as compared to each respective reference group. The prevalence of smear-positive PTB in the 39–59-year age group was 72.2%, (cOR 1.83, 95% CI 1.10, 3.06, *p* = 0.021), and 76.5% of female patients had DM (cOR 2.01, 95% CI 1.10, 3.69, *p* = 0.024). Female patients had three factors with lower prevalence and cOR for smear-positive PTB as compared to each respective reference group. Up to 48% of female patients worked as professionals and managers (cOR 0.33, 95% CI 0.14, 0.81, *p* = 0.015); 44% had middle-income level (cOR 0.40, 95% CI 0.18, 0.92, *p* = 0.030) and 37.5% were HCW (cOR 0.31, 95% CI 0.11, 0.88, *p* = 0.028). All these factors with *p* < 0.05 were used in the adjusted analyses to obtain the aOR.

**Table 3:** Characteristics of smear-positive PTB and simple logistic regression analysis among female PTB patients.

			Female patients		
Characteristic	Smear-positive PTB (*n* = 241) 49.4%	Smear-negative PTB (*n* = 133) 18.4%	Crude OR^c^ (95% CI)	χ^2^ (df)^a^	*p-*value
***Sociodemographic characteristics***	***n* (%)**	***n* (%)**			
**Age group (years)**					
18–38	108 (58.7)	76 (41.3)	1		
39–59	78 (72.2)	30 (27.8)	1.83 (1.10, 3.06)	5.322 (1)^b^	**0.021**
60–80	47 (65.3)	25 (34.7)	1.32 (0.75, 2.33)	0.936 (1)^b^	0.333
≥81	8 (80)	2 (20)	2.82 (0.58, 13.63)	1.654 (1)^b^	0.198
**Race**					
Malay	133 (61.3)	84 (38.7)	1		
Chinese	64 (65.3)	34 (34.7)	1.19 (0.72, 1.96)	0.464 (1)^b^	0.496
Indian	33 (73.3)	12 (26.7)	1.74 (0.85, 3.55)	2.292 (1)^b^	0.130
Other	11 (78.6)	3 (21.4)	2.32 (0.63, 8.54)	1.589 (1)^b^	0.207
**Residential location**					
Cheras	39 (57.4)	29 (42.6)	1		
Kepong	76 (71.7)	30 (28.3)	1.88 (0.99, 3.57)	3.761 (1)^b^	0.052
Lembah Pantai	53 (64.6)	29 (35.4)	1.36 (0.70, 2.63)	0.829 (1)^b^	0.363
Titiwangsa	73 (61.9)	45 (38.1)	1.21 (0.66, 2.21)	0.366 (1)^b^	0.545
***Socioeconomic characteristics***					
**Education level**					
No formal education	28 (71.8)	11 (28.2)	1.88 (0.86, 4.13)	2.485 (1)^b^	0.115
Primary school	14 (73.7)	5 (26.3)	2.07 (0.70, 6.12)	1.731 (1)^b^	0.188
Secondary school	130 (66.3)	66 (33.7)	1.46 (0.91, 2.32)	2.477 (1)^b^	0.115
Tertiary education	69 (57.5)	51 (42.5)	1		
**Type of residence**					
Low-cost	117 (68.4)	54 (31.6)	1		
Medium–high-cost	110 (59.8)	74 (40.2)	0.69 (0.44, 1.06)	2.858 (1)^b^	0.091
Other	14 (73.7)	5 (26.3)	1.29 (0.44, 3.77)	0.220 (1)^b^	0.639
**Occupation**					
Professionals and managers	12 (48)	13 (52)	0.33 (0.14, 0.81)	5.859 (1)^b^	**0.015**
Technicians, associate professionals and skilled workers	17 (56.7.)	13 (43.3)	0.47 (0.21, 1.09)	3.100 (1)^b^	0.078
Clerical, service, sales and support workers	48 (63.2)	28 (36.8)	0.62 (0.33, 1,16)	2.248 (1)^b^	0.134
Machine operators and elementary workers	3 (42.9)	4 (57.1)	0.27 (0.06, 1.28)	2.710 (1)^b^	0.100
Self-employed	29 (60.4)	19 (39.6)	0.55 (0.27, 1.13)	2.670 (1)^b^	0.102
Unemployed	49 (65.3)	26 (34.7)	0.68 (0.36, 1.28)	1.414 (1)^b^	0.234
Other	83 (73.5)	30 (26.5)	1		
**Income level (RM)**					
Low (≤3000)	230 (66.1)	118 (33.9)	1		
Middle (3001–13 148)	11 (44.0)	14 (56.0)	0.40 (0.18, 0.92)	4.713 (1)^b^	**0.030**
High (≥13 149)	0 (0)	1 (100)	0 (0)	0	1.000
***Clinical characteristics***					
**Smoking**					
Yes	11 (61.1)	7 (38.9)	0.86 (0.33, 2.28)	0.091 (1)	0.763
No	230 (64.6)	126 (35.4)	1		
**DM**					
Yes	52 (76.5)	16 (23.5)	2.01 (1.10, 3.69)	5.114 (1)	**0.024**
No	189 (61.8)	117 (38.2)	1		
**HIV**					
Yes	5 (62.5)	3 (37.5)	0.92 (0.22, 3.90)	0.013 (1)	0.908
No	236 (64.5)	130 (37.5)	1		
***Environmental characteristics***					
**Overcrowded household**					
Yes	25 (64.1)	14 (35.9)	0.98 (0.49, 1.96)	0.002 (1)	0.963
No	216 (64.5)	119 (35.5)	1		
**HCW**					
Yes	6 (37.5)	10 (62.5)	0.31 (0.11, 0.88)	4.807 (1)	**0.028**
No	235 (65.6)	123 (34.4)	1		

^a^Likelihood ratio (LR) test

^b^Wald test.

^c^Crude OR using simple logistic regression; bolded, *p* < 0.05; 1 = reference.

### The sex-related difference of factors associated with smear-positive PTB

Table 4 shows the final model for the factors associated with smear-positive PTB stratified by sex. Adjusted analyses showed that only three and two factors were associated with male and female smear-positive PTB patients, respectively. Male patients who worked as machine operators and elementary workers (aOR 2.23, 95% CI 1.24–4.02, *p* = 0.007) or who were self-employed (aOR 2.58, 95% CI 1.46–4.56, *p* = 0.001) had higher odds for having smear-positive PTB. Moreover, male patients who lived in ‘other’ residences (aOR 2.49, 95% CI 1.28–4.86, *p* = 0.007) and who were smokers (aOR 1.37, 95% CI 1.01–1.87, *p* = 0.045) also had higher odds for smear-positive PTB. Meanwhile, female patients with DM (aOR 1.92, 95% CI 1.05–3.54, *p* = 0.035) had higher odds for smear-positive PTB. Female patients who were HCW had lower odds for smear-positive PTB (aOR 0.33, 95% CI 0.12, 0.94, *p* = 0.039).

**Table 4:** Significant determinant factors of smear-positive PTB patients in Kuala Lumpur, the final model (*n* = 1121).

	Male			Female		
Characteristic	Adjusted OR^a^ (95% CI)	χ^2^ stat. (df)^b^	*p-*value	Adjusted OR^a^ (95% CI)	χ^2^ stat. (df)^b^	*p-*value
***Socioeconomic characteristics***						
**Occupation**				-	-	-
Professionals and managers	1.32 (0.58, 2.99)	0.433	0.510			
Technicians, associate professionals and skilled workers	1.09 (0.5, 2.26)	0.058	0.810			
Clerical, service, sales and support workers	1.51 (0.89, 2.57)	2.290	0.130			
Machine operators and elementary workers	2.23 (1.24, 4.02)	7.182 (1)^c^	**0.007**			
Self-employed	2.58 (1.46, 4.56)	10.576 (1)^c^	**0.001**			
Unemployed	1.44 (0.84, 2.46)	1.713 (1)^c^	0.191			
Other	1					
**Type of residence**				-	-	-
Low-cost	1					
Medium–high-cost	0.91 (0.65, 1.27)	0.327 (1)^c^	0.567			
Other	2.49 (1.28, 4.86)	7.238 (1)^c^	**0.007**			
***Clinical characteristics***						
**Smoking**				-	-	-
Yes	1.37 (1.01, 1.87)	4.026 (1)	**0.045**			
No	1					
**DM**	-	-	-			
Yes				1.92 (1.05, 3.54)	4.437 (1)	**0.035**
No				1		
***Environmental characteristics***						
**HCW**	-	-	-			
Yes				0.33 (0.12, 0.94)	4.282 (1)	**0.039**
No				1		

^a^Adjusted OR (male): for residential location, education level, type of residence, occupation, smoking status using the forward LR method

^a^Adjusted OR (female): age group, occupation, income level, DM status and HCW status using the forward LR method

^b^Likelihood ratio (LR) test

^c^Wald test; bolded, *p* < 0.05; 1 = reference.

## Discussion

### Prevalence of smear-positive PTB

Due to the growing number of TB cases over the years, the sex-related differences issues are worth highlighting and exploring them would be crucial [2]. Here, we reveal that the proportion of PTB is higher among males than in females. Overall, the prevalence of smear-positive PTB in the present study is lower compared with the national prevalence, which is 81.2% [2]. However, it is relatively higher compared to a study conducted in a general hospital in north Malaysia (Penang) (56.6%) [23], which is also an urban area in Malaysia. This could be due to the higher population density in Kuala Lumpur (7328 km^2^), which is almost five times higher compared to that of Penang (1692 km^2^) [17]. Studies from other countries such as Barcelona, Spain (26.4%) [24], Hyderabad city in Pakistan (28.3%) [9] and Almaty, Kostanay, and Kyzylorda city in Kazakhstan (34.3%) [25] have a relatively lower prevalence of smear-positive. Kuala Lumpur has achieved 100% urbanization with higher population density [17]; thus, overcrowding and poor living environments, with urban slum problems probably led to a higher prevalence of smear-positive PTB [26].

Our study highlights the sex-related differences in smear-positive PTB prevalence. Our findings show a higher prevalence of smear-positive PTB among males than females. This finding corroborates with numerous studies conducted in many countries [6, 7, 25, 26]. The M:F ratio was 1.4–4.6:1. However, other studies have also shown that the prevalence of smear-positive PTB among males could be lower than that in females, with ratios of 0.7:1 in Hyderabad [9] and Malakand [27], Pakistan.

These sex-related differences in PTB prevalence can be explained by behavioural and physiological variations [10]. The behavioural characteristics refer to epidemiological factors linked to sex-specific exposure to infection, such as risk behaviour (smoking, alcohol consumption, substance abuse), social roles (education level, type of occupation, income level) and dynamic transmission of the disease (overcrowding, poor ventilation, nosocomial setting) [10]. The physiological characteristics refer to the biological differences that determine the susceptibility to a given disease, such as X-linked genetics and the immune response [10].

### The sex-related difference of factors associated with smear-positive PTB

There is a scarcity of local studies focusing on the sex-related difference in factors associated with smear-positive PTB. The present study shows that, among male patients, occupation and type of residence were associated with smear-positive PTB. PTB cases are commonly associated with people with lower socioeconomic levels [26], and some workplaces or occupations may pose a higher risk for PTB due to environmental settings and the nature of the work itself. Our study reveals that male patients who were machine operators, elementary workers and who were self-employed had higher odds for smear-positive PTB.

Machine operators include factory workers in various manufacturing industries such as electrical and electronic products, textile and garment industries, rubber and oil palm processing and manufacturing, as well as motor vehicle drivers (i.e. bus and taxi drivers) [21]. The manufacturing industries are the second highest (22.8%) contributor to Malaysia’s economic growth and activity [17]. This sector presents excellent employment opportunities, especially for men, who are commonly the main breadwinners. In Malaysia, the manufacturing and construction industries, and waste management activities are dominated by men, who comprise 62%, 91.3% and 85% of workers in the manufacturing, construction and waste management sectors, respectively [28].

Hassan et al. revealed that the incidence of smear-positive PTB among garment factory workers in Dhaka, Bangladesh, was 1.75 per 1000 population, which is about 1.6 times more than the estimated national figure. They concluded that this could be due to overcrowded workplaces stemming from a paucity of floor space, and longer working hours [29]. Machine operators, which include bus drivers, are at higher risk for PTB, as the diesel emissions and carbon particles from asphalt in the air inhaled by the drivers could induce macrophage dysfunction and increase PTB susceptibility [30].

Elementary workers, such as municipal street cleaners [31] and construction workers [32], had a significantly higher risk for PTB. Municipal street cleaners commonly work without adequate protective equipment and are potentially exposed to different biohazards, including *MTb* [31]. Construction workers are generally exposed to high amounts of dust, such as crystalline silica, which can be a risk factor for respiratory dysfunction [33]. A study conducted in Iran among construction workers with moderate or severe silica exposure demonstrated that PTB prevalence was 3–4 times greater compared to that of the non-exposed workers [32]. Further, those who were self-employed, such as shop and market sales workers, had frequent contact with the community, which might expose them to PTB [34].

We observed that male patients who lived in residences categorized as ‘other’ (homeless, people living in a crowded environment such as shop lots in a commercial area, residence in shelters and prisons) had higher odds of having smear-positive PTB. Men who are homeless, with residence in shelters and a history of previous imprisonment have higher rates of PTB [6]. A local study among prison inmates indicated that 7.7% of them had undiagnosed active PTB. Of these, 17.7% was smear-positive PTB [16]. As the male sex dominates the majority of homeless people in Kuala Lumpur [35] and prisons (93.2% in 2017) [36], we postulated that this could have led to the significant association with smear-positive PTB observed in the present study. Furthermore, socioeconomically deprived homeless people could have low body immunity due to poor nutrition and increased susceptibility to disease [26]. Overcrowding, inadequate ventilation, and prolonged cell confinement are all factors conducive to *MTb* transmission [37].

The present study shows that being a smoker is associated with smear-positive PTB among male patients. A study conducted in Iran found that men who are smokers have 98% increased risk of developing smear-positive PTB [7]. In comparison, men who are non-smokers have only 5% increased risk of developing smear-positive PTB [7]. Cigarette smoking induces pathophysiological changes in the respiratory system, which weakens the respiratory tract immunity, which can promote *MTb* growth [7]. Matsumoto et al. found that the chest radiographs of smear-positive PTB male patients who were current smokers showed lung cavities [38]. Lung cavitation is linked to a very high bacillary load and may delay sputum conversion, thus increasing infection transmission [3]. Transmission could be higher among men, probably due to the proportion of male current smokers being 30 times higher compared to females [39]. Thus, smoking is a crucial modifiable risk factor of TB, and emphasis on the importance of tobacco regulation could not be stronger.

Our study demonstrates that female patients with DM had higher odds of having smear-positive PTB. This could be due to the high overall prevalence of DM in Malaysia (17.5%), with higher prevalence among women (18.3%) compared to men (16.7%) [39]. DM adversely affects the expression of complement receptor 3–positive monocytes and interleukin-2 receptor expression, together with decreased lymphocyte proliferation, which leads to suppressed immunity and diminished ability to contain the organism [40]. Moreover, TB has been associated with poorly controlled DM [41]. As previous studies have demonstrated that women had poorer DM control compared to men [42], this could explain the association of DM with smear-positive PTB among the female patients in our study, which requires further evaluation. On the contrary, Abedi et al. revealed no association between DM and sex in development of smear-positive PTB [7]. This could be due to the relatively lower overall DM prevalence in Iran (11.6%), with an almost equal proportion of DM among men (11.1%) and women (12.1%) [43].

Our study shows that female patients who are HCW had lower odds for smear-positive PTB, which is contrary to previous findings that indicated that HCW workers are at higher risk for TB [15]. This could be due to the fact that previous studies conducted among HCW diagnosed with TB were not stratified by sex. We postulate that the lower odds for smear-positive PTB might be due to the better TB preventive knowledge [44], regular training, personal protective equipment, annual Mantoux testing and PTB symptom screening provided to HCW, which warrants further investigation.

The limitations of our study include the cross-sectional nature of the study design, where causal inference cannot be made. The status of crowdedness was based on estimation by the authors by comparing the type of residence and the number of inhabitants. The sex-related differences findings from this study also need to be interpreted by each sex and with caution, as the objective was not to make a pair-wise comparison between both sexes. Furthermore, the sample size calculated was based on overall PTB rather than each stratum, which rendered smaller sample size among the female patients. Nevertheless, the findings tried to highlight the different associated factors among males and females since the authors believe that it is important for future planning to be focus based on sex-based assessment after addressing all the limitations. Although most of the discussion refers to previous studies focusing on PTB, we suggest that this could be the basis for the association with smear-positive PTB among the patients in the present study, where the mechanism warrants further studies. Our study could fill some gaps in knowledge regarding the sex-related differences in smear-positive PTB and its associated factors in our population. These findings could be the baseline for further prospective studies.

## Conclusion

The prevalence of smear-positive PTB is higher in males compared to females. Occupation, type of residence and smoker status are associated with smear-positive male PTB patients. Female patients with DM are associated with smear-positive PTB, while female patients who are HCW have lower odds for smear-positive PTB. The current TB control program, especially on smear-positive PTB likely requires strategizing and stratification by sex.

## Abbreviations

AFB: Acid-Fast Bacilli
aOR: Adjusted OR
cOR: Crude odds ratio
CI: Confidence interval
DM: Diabetes Mellitus
HCW: Healthcare Worker
HIV: Human Immunodeficiency Virus
*Mtb*: Mycobacterium Tuberculosis
M:F: Male to female
TBIS: National Tuberculosis Information System
PTB: Pulmonary Tuberculosis
RM: Ringgit Malaysian
SPSS: Statistical Package for Social Sciences
SD: Standard Deviations
TB: Tuberculosis
WHO: World Health Organization
